# Outcomes after Transcatheter Mitral Valve Implantation: A Literature Review

**DOI:** 10.3390/jpm12122074

**Published:** 2022-12-16

**Authors:** George Samanidis, Meletios Kanakis, Konstantinos Perreas

**Affiliations:** 1 Department of Adult Cardiac Surgery, Onassis Cardiac Surgery Center, 356 Leoforos Syggrou, 17674 Athens, Greece; 2 Department of Pediatric and Congenital Heart Surgery, Onassis Cardiac Surgery Center, 356 Leoforos Syggrou, 17674 Athens, Greece

**Keywords:** transcatheter, mitral valve, replacement, implantation

## Abstract

Mitral valve disease is the most common heart valve disease worldwide. Surgical mitral valve replacement or repair has been an established therapy in patients with severe mitral valve disease for many years. On the other hand, many patients with advanced mitral valve disease and severe comorbidities are treated conservatively and are excluded from the surgical procedure. Furthermore, in patients with severe comorbidities, transcatheter mitral valve repair by edge-to-edge technique with MitraClip or transcatheter mitral valve repair with a non-absorbable ring have been added as therapeutic options over the last few years. Alternative procedures for the treatment of patients with advanced prosthetic or native mitral valve diseases include transcatheter access for replacement or implantation of a new prosthetic valve in the diseased mitral valve. Promising results were published about short-term outcomes of patients who underwent the transcatheter mitral valve replacement. The current view and results of the transcatheter mitral valve implantation in patients with advanced native or prosthetic mitral valve disease are briefly discussed.

## 1. Introduction

Mitral valve disease (MVD) is the most common heart valve disease (HVD) worldwide [1,2,3]. In developed countries, the dominant MVD are primary and secondary mitral regurgitation, while in developing and other countries it is rheumatic disease. In patients with untreated mitral valve pathology, the progressive worsening of left ventricular dysfunction and development of fixed pulmonary hypertension affect morbidity and mortality [1,2,3]. Surgery remains the treatment of choice for symptomatic mitral valve regurgitation (MR), mitral stenosis (MS) and mixed mitral valve disease (MMVD) [1,2,3]. Surgical mitral valve replacement (sMVR) in patients with MS or MMVD has been an established technique for many years, while surgical mitral valve repair (MVr) is suggested as the gold standard operative technique for patients with MR [1,2]. Many patients with advanced MVD and severe comorbidities such as previous cardiac surgery are treated conservatively and are excluded from the surgical procedure. This conservative approach may affect the survival rate of these patients. An alternative procedure for MVD treatment has been proposed and includes the transcatheter access of the mitral valve for repair or implantation of theprosthetic valve device in the diseased mitral valve (MV) [4,5,6,7]. Patients directed to MVr by edge-to-edge technique with mitraclip or transcatheter MVr with a non-absorbable ring constitute the largest population for these aforementioned techniques and patients directed to transcatheter mitral valve implantation (TMVI) represent the minority [7,8,9,10]. Nowadays, controversial results have been reported about short- and mid-term results of patients who underwent the TMVI [8,9,10]. Moreover, the off label indication for TMVI is the degeneration of the bio-prosthetic mitral valve. Degenerated bio-prosthetic mitral valves will be another challenge for the therapeutic approach in the future due to the increasing number of patients who underwent sMVR in the past [9,11,12]. First TMVI was reported in 2012 with CardiAQ valve (Edwards Life Sciences, Irvine, California, United States) [13]. Nowadays, a significant number of new mitral devices are tested for durability and effectiveness during follow-up.

## 2. Methods

This review evaluates the current challenges of transcatheter mitral valve replacement or implantation in patients with advanced mitral valve disease. PubMed was used for searching publications regarding the outcomes of patients after TMVI. The chronic period for data extraction was 2013–2022. This review includes studies with reported outcomes after TMVI. ‘’Transcatheter mitral valve implantation’’ or ‘’transcatheter mitral valve replacement’’ and ‘’outcomes’’ were used as key words for searching on the PubMed site. The perioperative morbidity and mortality reports after TMVI were outcomes of interest. However, the TMVI procedure is a relevant new technique for mitral valve disease treatment and minimal data are available for safe analysis.

## 3. Results

### 3.1. Indication for Transcatheter Mitral Valve Implantation

The most common indication for TMVI is advanced MVD due to severe secondary MR with reduced left ventricle ejection fraction (LVEF) and heart failure (HF), severe mitral annulus or mitral leaflet calcification, severe mixed mitral valve pathology with small mitral valve orifice area, degenerated bio-prosthetic mitral valve disease and failed MVr (ring annuloplasty) [14,15]. In addition, patients with severe comorbidities and high operative risk are considered as candidates for TMVI [14,15]. Patients with symptomatic primary MR and asymptomatic MR with left ventricle (LV) dysfunction are the ideal candidates for surgical MVr, while patients with secondary moderate to severe and symptomatic MR are candidates for various treatment modalities such as medical treatment alone, transcatheter repair (edge-to edge technique, repair), and transcatheter repair with medical treatment to SMVR [1,2,3]. The recent studies demonstrated that outcomes (hospitalization and mortality rate during 1 and 2 years of follow-up) of patients with secondary MR who were treated by transcatheter MVr and medical treatment did not differ compared to the medical treatment only [16,17]. On the other hand, Stone et al. reported that the transcatheter MVr had a lower rate for hospitalization and all-cause mortality within 2 years versus the medical treatment alone [18]. 

### 3.2. Methods for Transcatheter Mitral Valve Implantation

Two methods for the catheter-based mitral valve implantation have been proposed over the last few years, and these are the transapical and transfemoral/transseptal approach [13,15]. In both techniques, the prosthetic valve is implanted in the native or prosthetic pathological mitral valve under guidance of the transesophageal echocardiography (TEE), ideally in a hybrid room. Transfemoral access is considered a less invasive technique due to lower peri-procedural complications and mortality rates than the transapical access [19,20]. The transfemoral access is achieved by puncturing the common femoral vein under guidance of ultrasonography and finally the insertion of the large diameter sheath for the delivering system of the prosthetic mitral valve [15,16,20]. On the other hand, the transapical implantation needs a small left anterior thoracotomy and small incision in the LV for inserting the delivery system of prosthetic valve through the LV apex with the subsequent implantation of the prosthetic valve in the mitral annulus [21]. The most common prosthetic mitral valves, which have been reported for TMVI are the CardiAQ-Edwards TMVR System (Edwards Lifesciences, Irvine, CA, USA), EVOQUE TMVR System (Edwards Lifesciences, Irvine, CA, USA), SAPIEN M3 System (Edwards Lifesciences, Irvine, CA, USA), Cardiovalve TMVR system (Cardiovalve Ltd., Or Yehuda, Israel), Tiara TMVR System (Neovasc Inc., Richmond, BC, Canada), Tendyne Mitral Valve System (Abbott Vascular, Roseville, MN, USA), INTREPID TMVR System (Medtronic, Inc., Redwood City, CA, USA), Caisson TMVR system (LivaNova PLC, London, UK), and HighLife TMVR system (HighLife Medical, Paris, France) [21,22,23]. The early results after TMVI demonstrated the promising short-term results regarding early morbidity and 30-day mortality, while the mid- and long-term results after implantation are expected in the next 3–5 years [19,20,21,22,23]. Long-term durability of devices, left ventricle outflow tract (LVOT) obstruction during or after procedure, and early and late prosthetic valve thrombosis after implantation are the most common difficulties and complications that should be solved by manufacturers, researchers, and clinicians [14,20,21,22,23]. New prosthetic valves and delivered devices for TMVI have been developed by many institutions and manufacturers worldwide with promising results [23]. At this time, advantages and disadvantages of the prosthetic valve, which is used in TMVI, is difficult to present due to the limited implanted prosthetic valves and limited number of patients who treated with these techniques. About other valves, minimal data are available about the routine practice for using these valves, due to the limited number of valves that were implanted. Some results after the TMVI implantation is available in patients who were received Sapien (balloon-expandable) and Tendyne (self-expanding) valves. On the other hand, these cannot be compared in practice because the implantation technique is different between the two valves. For implanting, the Tendyne valve needs a mini right thoracotomy and it is a different philosophy from the percutaneous valve implantation, which is used in the Sapien valve. Although Tendyne is included in TMVI, the delivery system of the Tendyne valve is inserted via a small incision in the left ventricle apex. In practice, it is a different approach from the percutaneous valve implantation. Furthermore, in the Sapien, the valve implantation is used knowing the delivery system such as in the TAVI, and it is for the off-label use for the prosthetic mitral valve degeneration pathology and in case with MVr failure or advanced mitral annulus calcification (MAC). Moreover, the second study with some short- and mid-term results was presented after the Intrepid valve implantation, and it regarded only 50 patients. Mid- and long-term outcomes of the current development clinical trial are expected to confirm the preliminary acceptable short-term results after TMVI, which have been announced in published materials in the past.

### 3.3. Outcomes of Patients after Transcatheter Mitral Valve Replacement or Implantation

Most of the patients that underwent TMVI have severe comorbidities, which affect the length of stay in hospitals and the ICU. In-hospital mortality and all-cause mortality are also increasing due to more advanced cardiac pathologies (coexisting heart failure, permanent atrial fibrillation, pulmonary artery hypertension and previous cardiac surgery). Transapical (TA) mitral valve implantation is the newest TMVI technique and is used in patients with a high risk for operation. The published results are encouraging regarding mortality and morbidity. On the other hand, most of these patients included in these studies were patients with severe comorbidities with a high or prohibitive risk for surgical intervention. Studies with a large study population and long-term outcomes are limited. Furthermore, many transcatheter devices for TMVI are in a preclinical study evolution or in the study design. The recruitment of patients for TMVI plays a crucial role to increase the number of patients who will undergo TMVI in order to derive any safe conclusions about the effectiveness and long-term durability of these devices. On the other hand, the valve-in-valve implantation in the degenerated prosthetic valve or ring annuloplasty was reported in the literature with acceptable short- and mid-term results [9,11,12]. In this review, we present the current studies with reported outcomes after TMVI in the last few years. We present in Table 1 the baseline characteristics, indications and the most common post-procedural complications from these studies, while in Table 2, the outcomes of patients who underwent TMVI are presented.

Recently, the Tendyne Mitral Valve system for TMVI has received a CE mark for the treatment of patients with MV pathologies. Wild et al. reported results from a multicenter study, which included 108 surgical high risk patients with symptomatic MV treated by the Tendyne Mitral Valve system [24]. The majority of patients were readmitted to the hospital due to heart failure and most of them had pre-procedure NYHA class III-IV. The authors reported that the in-hospital mortality was 8%, in-hospital cerebrovascular events were 3%, major bleeding was 11%, valve thrombosis was 1%, permanent pacemaker (PPM) implantation was 2%, sepsis was observed at 10% and acute kidney injury requiring dialysis was 5%. Two patients died peri-procedurally. A total of 64 (66%) patients were discharged at home, with the remaining being transferred to other hospitals or rehabilitation centers [24]; meanwhile, the 30 day mortality was 12%. In addition, the authors reported that during the median follow-up of 50 days, 73% of patients were NYHA functional class I or II (*p* < 0.001), as this compared to pre-TMVI. Gössl et al. published the results after the same mitral valve device implantation in 20 patients [25]. No procedural mortality was reported, while the 30-day mortality and the one-year all-cause mortality was 5% and 40%, respectively. During the one-year follow-up, six patients (31%) were re-admitted for hospitalization due to HF, and the NYHA functional class was upgraded in 11 patients who were alive after one year. Muller at al., in 2021, presents the outcomes after the Tendyne mitral valve implantation at 2 years [26]. This multicenter study included 100 patients with the severe MR of native MV, and the study period was 2014–2017. The study was a clinical trial titled, ‘’Expanded Clinical Study of the Tendyne Mitral Valve System’’. At the one- and two-year follow-up, the all-cause mortality was 26% and 39%, respectively, while the post-procedural neurological complications, including TIA and stroke, at 1- and 2-year follow-ups, were 6% and 9%, respectively. PPM implantation incidence at the 1- and 2-year follow-up was 7% and 8%, respectively. The thrombosis of the prosthetic valve was observed in 6% of patients during the 2-year follow-up. The most common device related to an adverse event during the 2-year follow-up was the paravalvular leak in 9%. 

CardiAQ-Edwards TMVR System was another prosthetic mitral valve, which was used for the treatment of advanced MV pathologies via the transfemoral approach [27]. Ussia et al. presented their results after a first and second-generation CardiAQ mitral valve bioprosthesis implantation in a patient with severe MR. The patient survived 30-days after the implantation at NYHA class I. In 2016, Sondergaard et al. presented their results after CardiAQ implantation in three patients, and one of them died in hospital [28].

The first in-man implantation, the Tiara TMVR system, was described by Cheung et al. in 2014 [35]. Ludwig at al. presented their results after implantation with the following two devices, the Tendyne TMVR system and Tiara™ [29]. In four patients, the Tiara prosthetic valve was implanted and no procedural or in hospital mortality was observed. Stroke, prosthetic valve thrombosis, myocardial infarction or re-intervention was not observed after 30 days. In this report, the authors demonstrated that the mortality rate after 3, 6 and 12 months was 10, 22.2 and 33.3%, respectively, but unfortunately they did not focus on mortality rate with regards to the specific type of mitral valve prosthesis. The clinical trial, the Tiara™ Transcatheter Mitral Valve Replacement Study (TIARA-II), was started in 2017, and the estimated date for completion will be in 2026. 

Bapat et al. presented the early experience after the Medtronic Intrepid™ Transcatheter Mitral Valve Replacement System implantation in 50 patients in the context of the Intrepid Global Pilot Study Investigators [30]. Most of the patients had secondary MR (72% of patients). The prosthetic valve was implanted transapically. A successful implantation was achieved in 98% of patients. The median procedure time was 100 min and in 5% of patients an intra-aortic balloon pump was placed. The median follow-up of patients was 173 days, and the 30-day mortality was 14%. The all-cause mortality during follow-up was 9.8%. After procedure (>30 days), the rehospitalization for heart failure was recorded in 19.5% of patients. Reoperation for bleeding immediately after operation was performed in five patients, while postoperative neurological complications after implantation and during follow-up (including stroke) were observed in 6.4% of patients. During the 30-day follow-up, the NYHA class was upgraded for the functional class I and II in 79% of patients. The pulmonary artery systolic pressure was reduced post-procedural (*p* < 0.001). Zahr et al. reported the 30-day outcome of 15 patients, who were treated for moderate to severe or severe MR by the Intrepid™ Transcatheter Mitral Valve Replacement System and transfemoral approach [31]. The study period was from 2020 to 2021 and the 35-F-delivered system was used for the transfemoral valve implantation. The patient clinical status and echocardiography were followed up at 30-days. The median age of the study population was 80 years old, and 53% of the patients have undergone cardiac surgery in the past. The preoperative primary MR was recorded in 67% of patients. No deaths, postoperative neurological complications, re-interventions and PPM implantation was observed at the 30-day post-procedural follow-up period, while major or worse bleeding was recorded in 47% of patients. In 15% of patients, moderate LVOT obstruction was observed. 

The first in-human implantation outcomes with the new TMVI devices (Edwards Sapien M3, Edwards Lifesciences, Irvine, CA, USA) presented by Webb et al. [32]. The prosthetic valve was implanted by the transfemoral approach. The study period was from 2017 until 2018 and 10 patients were included. The mean age was 76.1 years old and 50% of the patients were men. Degenerative and secondary MR was observed in 40 and 40% of patients, respectively. The mortality and stroke in the first 30 days after implantation was 0%. LVOT obstruction was not recognized clinically or echocardiogrphically during or after implantation. 

The transcatheter mitral valve implantation in patients with the degenerated prosthetic valve and previously failed surgical repair is the most common practice for prosthetic valve implantation by transcatheter methods. In addition, these patients are poor candidates for reoperation due to a high perioperative risk for complication and deaths [19,33,34,36]. Eleid et al. presented a study of the early outcomes (1-year) of a multi-center study of 87 patients who underwent TMVI for failed mitral bioprothesis in 60 patients [valve-in-valve (VIV)], ring annuloplasty [valve-in-ring (VIR)] and severe mitral annular calcification [valve in mitral annular calcification (VMAC)] in 60, 15 and 12 patients, respectively [33]. The study period was 2014–2017 and the balloon-expandable SAPIEN, SAPIEN XT, or SAPIEN 3 THV (Edwards Lifesciences, Irvine, California) were used for TMVI. The mean age of patients was 75 years old, and procedural success was 90%. TMVI was performed by trans-septal/transfemoral and transapical methods in 84 and 3 patients, respectively, while the total peri-procedure mortality was 5%. The LVOT obstruction was observed in 9% of patients and most frequently occurred in the valve-in ring (20%) group. Prosthetic valve thrombosis was diagnosed in two patients. The 30-day mortality overall was 6%. The mean follow-up in the VIV and VIR was 283 and 309 days, respectively. The survival rate at the 1-year follow-up in VIV, VIR and VMAC were 86%, 82% and 57%, respectively. The predictor factor for the LVOT obstruction was a higher LVEF and most of the patients were treated conservatively. Guerrero et al. reported 30-day outcomes of TMVI for patients who underwent VIV, VIR and VMAC [34]. The study period was 2013–2017. It was a retrospective analysis of the national database of USA and included 903 patients from 127 hospitals. Most of these patients underwent VIV = 680 patients, and SAPIEN 3 valve (Edwards Lifesciences, Irvine, CA) was the most common prosthetic valve. The 30-day mortality in the study population was 10.1% and it was higher in the VMAC group (21.8%). The accesses for TMVI were transapical and transseptal methods in 44.8% and 43.1%, respectively. IABP insertion was needed in 3.2% of overall patients, while the PPM insertion was 1.2%. The incidence of postoperative neurological complications at the 30-day follow-up was 1.7% and myocardial infarction was 0.5%. Moreover, knowledge about TMVI in patients with failed mitral bioprothesis was reported by Whisenant et al. [19]. Their research focused on 1-year outcomes of patients who underwent VIV for failed mitral prosthesis. The SAPIEN 3 valve (Edwards Lifesciences, Irvine, CA, USA) was used for TMVI. The study period was from 2015 until 2019 and included patients. The transseptal approach was the most common access for TMVI (86.7%). The patients’ age was 73.3 years old and prosthetic mitral valve stenosis was the most common pathology (55.4%). The most commonly implanted SAPIEN 3 size was 29-mm (56%). At 30 days, the all-cause mortality, stroke and PPM implantation were 5.4%, 1.1% and 1.4%, respectively. Meanwhile, during the 1-year follow-up, the all-cause mortality, stroke, PPM implantation, mitral valve re-intervention and device thrombosis were 16.7%, 3.3%, 2.1%, 0.8% and 0.5%, respectively. 

## 4. Conclusions

Surgical mitral valve replacement or repair remain the gold standard technique for patients with primary MR therapy, while the patients with secondary MR, severe MAC, degenerated prosthetic mitral valve and failed MVr (surgically or transcatheter) remain in the gray zone regarding the appropriate choice for invasive or non-invasive therapy. The rising numbers of elderly patients with coexisting complex comorbidities with advanced mitral valve pathologies creates a large population not suitable for surgical operation. Untreated progressive mitral valve diseases negatively affect the quality of life, morbidity and survival rate of these patients. Alternative options for therapy are transcatheter mitral valve therapy, including the edge-to-edge technique, percutaneous mitral ring annuloplasty and TMVI. Meanwhile, in patients with secondary (functional) moderate to severe or severe MR, the MitrClip have promising results; the patients with severe MAC, degenerated mitral bioprothesis and severe primary MR with LV dysfunction consist of practice-inoperable patients, and probably need to be treated conservatively. TMVI can cover the gap for making a decision for an appropriate therapy in these patients. Although the 30-day mortality after TMVI is 0–10%, more studies with larger-studied populations are required in order to derive safe conclusions about the effectiveness of TMVI therapy and the durability of new devices in current clinical practice. 

## Figures and Tables

**Table 1:** Baseline characteristics of patients, indications for TMVI and the most common post-procedural complications after TVMI. VIV = valve-in-valve; VIR = valve-in-ring; VIM = valve-in-mitral valve calcification; N/A = not applicable; MAC = mitral annular calcification; MR = mitral valve regurgitation; MS = mitral stenosis; MD = mixed disease; NYHA class = New York Heart Association Classification; IQR = interquartile range.

Authors	Device	Study Population, Number of Patients	Age, Years Old	Gender (Female), %	Pre-Procedural NYHA Class III or III–IV, %	Indications	Post-TMVI Cerebrovascular Accident, %	Post-TMVI Permanent Pacemaker Implantation, %	Success Implantation, %
Wild et al. [24]	Tendyne™	108	Mean 75.5 ± 7	43	86	Severe MR	N/A	N/A	96
Gössl et al. [25]	Tendyne™	20	Mean 78 ± 6	55	90	MR in11 pts MAC in 9 pts	5	N/A	95
Muller et al. [26]	Tendyne™	100	Mean 74.7 ± 8.0	31	66	Sever MR	N/A	N/A	97
Ussia et al. [27]	CardiAQ™	1	72	0	100	Severe MR	0	0	100
Sondergaard et al. [28]	CardiAQ™	3	Mean 82.3	33	100	Severe MR	0	N/A	100
Ludwig et al. [29]	Tendyne™ and Tiara™	7 and 4	Mean 73.4	72.7	100	Severe MR	0	0	100
Bapat et al. [30]	Intrepid™	50	Mean 73 ± 9	42	86	Severe MR	4	0	98
Zahr et al. [31]	Intrepid™	15	Median 80 (IQR:73–84)	13	67	Severe MR	0	0	93.3
Web et al. [32]	Sapien M3	10	Mean 76.1 ± 5.5	50	100	Severe MR	0	0	100
Eleid et al. [33]	Sapien, Sapien XT, Sapien 3 THV	Total = 87 VIV = 60 VIR = 15 VIM = 12	Mean 75± 11 72 ± 8 79 ± 9	57 60 42	100	Sever MR and MAC	N/A	N/A	Overall = 90
Guerrero et al. [34]	Sapien, Sapien XT, Sapien3	Total = 903 VIV = 680 VIR= 123 VIM = 100	Median 76 73 77	Overall = 59 59.9 48 69	89	Sever MR and MAC	1.9	1.2	Overall = 78.7
Whisenant et al. [19]	Sapien 3 THV	1529 (VIV)	Mean 73.3 ± 11.8	59.1	86	Prosthetic valve MS, MR, MD	0.7	0	96.8

**Table 2:** **Studies with outcomes during follow-up included in review**. VIV = valve-in-valve; VIR = valve-in-ring; VIM = valve-in-mitral valve calcification; N/A = not applicable.

Authors	Device	Study Period, Years	Study Population, Number of Patients	In-Hospital Mortality, %	1-Year Mortality, %	2-Years Mortality, %
Wild et al. [24]	Tendyne™	2020–2021	108	12	N/A	N/A
Gössl et al. [25]	Tendyne™	2018–2019	20	5	40	N/A
Muller et al. [26]	Tendyne™	2014–2017	100	6	26	39
Ussia et al. [27]	CardiAQ™	2016	1	0	N/A	N/A
Sondergaard et al. [28]	CardiAQ™	2015	3	33	N/A	N/A
Ludwig et al. [29]	Tendyne™ and Tiara™	2016–2020	7 and 4	0	33	N/A
Bapat et al. [30]	Intrepid™	2018	50	14	24	N/A
Zahr et al. [31]	Intrepid™	2020–2021	15	0	N/A	N/A
Web et al. [32]	Sapien M3	2017–2018	10	0	N/A	N/A
Eleid et al. [33]	Sapien, Sapien XT, Sapien 3 THV	2014–2017	87 (VIV, VIR, VIM)	6	32 (VIM) 14 (VIV, VIR)	N/A
Guerrero et al. [34]	Sapien, Sapien XT, Sapien3	2013–2017	903 (VIV, VIR, VIM)	18 (VIM) 6.3 (VIV) 9 (VIR)	N/A	N/A
Whisenant et al. [19]	Sapien 3 THV	2015–2019	1529 (VIV)	5.4	16.7	N/A

## Data Availability

Not applicable.
